# Rainbow trout exposed to benzo[a]pyrene yields conserved microRNA binding sites in DNA methyltransferases across 500 million years of evolution

**DOI:** 10.1038/s41598-017-17236-x

**Published:** 2017-12-04

**Authors:** Christopher Kuc, Daniel J. Richard, Samantha Johnson, Leslie Bragg, Mark R. Servos, Andrew C. Doxey, Paul M. Craig

**Affiliations:** 1 Department of Biology, University of Waterloo, Waterloo, ON Canada; 2 Molecular and Cellular Biology, University of Guelph, Guelph, ON Canada; 3 Ontario Veterinary College, University of Guelph, Guelph, ON Canada

## Abstract

The objective of this study was to examine the regulation of DNA methylation following acute (24 h) and prolonged (14 d) exposure to low (1 ng/L) and high (10 ng/L) benzo[a]pyrene. However, with the recent release of the rainbow trout genome, we were able to conduct a more detailed analysis regarding the regulation of the enzymes involved in DNA methylation; DNA methyltransferases (DNMTs). Bioinformatic approaches were used to identify candidate microRNA (miRNA) that potentially bind to the *DNMT1* and *DNMT3a* 3′UTR. Results indicated a significant decrease in global methylation in both liver and muscle, with an associated decrease in DNA methyltransferase activity and *DNMT3a* transcript abundance. There was a significant increase in one specific candidate miRNA (miR29a) that was predicted to bind to *DNMT3a*. Taking a comparative genomics approach, the binding sites of miR29a to the *DNMT3a* 3′UTR was compared across species, spanning fish to mammals, and revealed a highly conserved binding motif that has been maintained since the vertebrate ancestor, approximately 500 million years ago. This research establishes that miRNA act as an essential mediator between the environment and DNA methylation patterns via DNMTs, which is further confirmed by a genomic regulatory mechanism that has been deeply conserved throughout evolution.

## Introduction

DNA methylation is a well-established epigenetic process wherein DNA methyl transferases (DNMTs) transfer a methyl group to cytosine residues adjacent to guanines (CpG sites) in the promoter region of specific genes. By inhibiting the binding of regulatory regions by transcription factors, DNA methylation is typically associated with gene silencing^1^, although recent evidence suggests methylation can enhance transcription factor binding^2–4^. However, it is accepted that alterations in the functionality of DNMTs can lead to global instability in transcription rates and contribute to numerous pathological conditions including cancer^5,6^. Methylation patterns are established through several different DNMTs, two of which are of interest to this study: DNMT1 and DNMT3a. DNMT1 is termed the maintenance DNMT as it interacts with hemi-methylated DNA^7^. During DNA replication, DNMT1 is localized to the replication fork where it copies the methylation patterns to the newly synthesized strand, thus maintaining the methylation profile^8^. Additionally, DNMT1 repairs DNA methylation on strands that have lost their methyl group^9^. In contrast to DNMT1, DNMT3a is termed a *de novo* DNMT^7^, and functions by methylating CpG sites in DNA, which ultimately leads to gene silencing^10^. While DNMTs are essential in regulating methylation patterns in DNA, recent studies have suggested that other epigenetic mechanisms play an essential, tandem role in DNA methylation.

Many recent studies have suggested that a variety of microRNAs (miRNA) specifically target DNMTs in leukemia^11^, breast cancer^12^, bronchopulmonary dysplasia^13^, and liver fibrosis^14^, which accentuate the pivotal role of miRNA in disease progression and pathology. MicroRNA are short (~20–22nt), non-coding RNA that exert a pleiotropic effect on multiple targeted transcripts through both normal and pathological conditions within the cell^15^. The evolution and conservation of miRNA are well established in the vertebrate lineage, and studies have suggested that the expansion in the number of miRNA families are strongly associated with the evolution of organismal diversity and complexity^16–19^. However, while conservation in miRNA sequence persists in vertebrates, miRNA target site conservation in the 3′UTR is poorly understood. Several studies suggest that the 3′UTR are under selective pressures to maintain complementarity to the corresponding miRNA^20,21^, while others have demonstrated a rapid evolution in target sites^22^. More recently, Xu *et al*.^23^ demonstrated that while target sites are evolutionarily conserved in a given species, this conservation is progressively lost in a step-wise manner through expanding taxonomic groups. This suggests that similar phenotypic responses in species to environmental perturbation have evolved both distinct and comparative mechanism of transcriptional regulation, and caution needs to be taken when comparing miRNA and target regulation in mammalian literature versus more distantly related species, such as teleosts.

Recent evidence demonstrates that Benzo(a)pyrene (B[a]P) plays a significant role in the hypo-methylated state of DNA, both global and specific, in mammalian and teleost models^24,25^, and attribute these changes to the repression in several DNMT expression patterns. B[a]P is a polycyclic aromatic hydrocarbon (PAH) formed by the incomplete combustion of organic material and is found ubiquitously in the aquatic environment^26^. It is an established carcinogen and has been linked to multigenerational effects through alterations in DNA methylation patterns^27,28^. Further, there are numerous studies confirming a variety of miRNA that respond to B[a]P exposure, both in mammalian cell studies^29^ and *C. elegans* ^30^. It is therefore ideal to understand the molecular mechanisms by which B[a]P exposure leads to global changes in the methylation, and the potential regulatory mechanisms of methylation patterns through miRNA.

In this study, we used a multidisciplinary approach involving enzymatic analysis, quantitative PCR, and *in silico* bioinformatic analysis to investigate the interplay between DNMTs, miRNA, and their conserved binding sites in rainbow trout exposed to B[a]P. First, using rainbow trout (*Oncorhynchus mykiss*), we examined the impact of acute and chronic B[a]P exposure on the global methylation pattern and DNMT activity in the liver, as this is the major site of B[a]P detoxification^26^. Second, mining the recently published rainbow trout genome^31^ and miRNA transcriptome^32^, we predicted miRNAs targeting the 3′UTRs of DNMT1 and 3a, which were then experimentally quantified using quantitative PCR. Lastly, given the fundamental importance of DNMT involvement in vertebrate epigenetic regulation during stress and disease^33,34^, we examined the degree to which the predicted miRNA binding are conserved throughout vertebrate genomes. We demonstrate that a specific miRNA (miR-29) is elevated upon B[a]P exposure, and identify ultra conserved miR-29 binding sites in the DNMT3A 3′UTR. Our work reveals a conserved regulatory mechanism connecting environmental stress and DNA methylation via DNMTs.

## Results

### B[a]P exposure alters global methylation levels and DNMT3a expression

**Figure 1:**
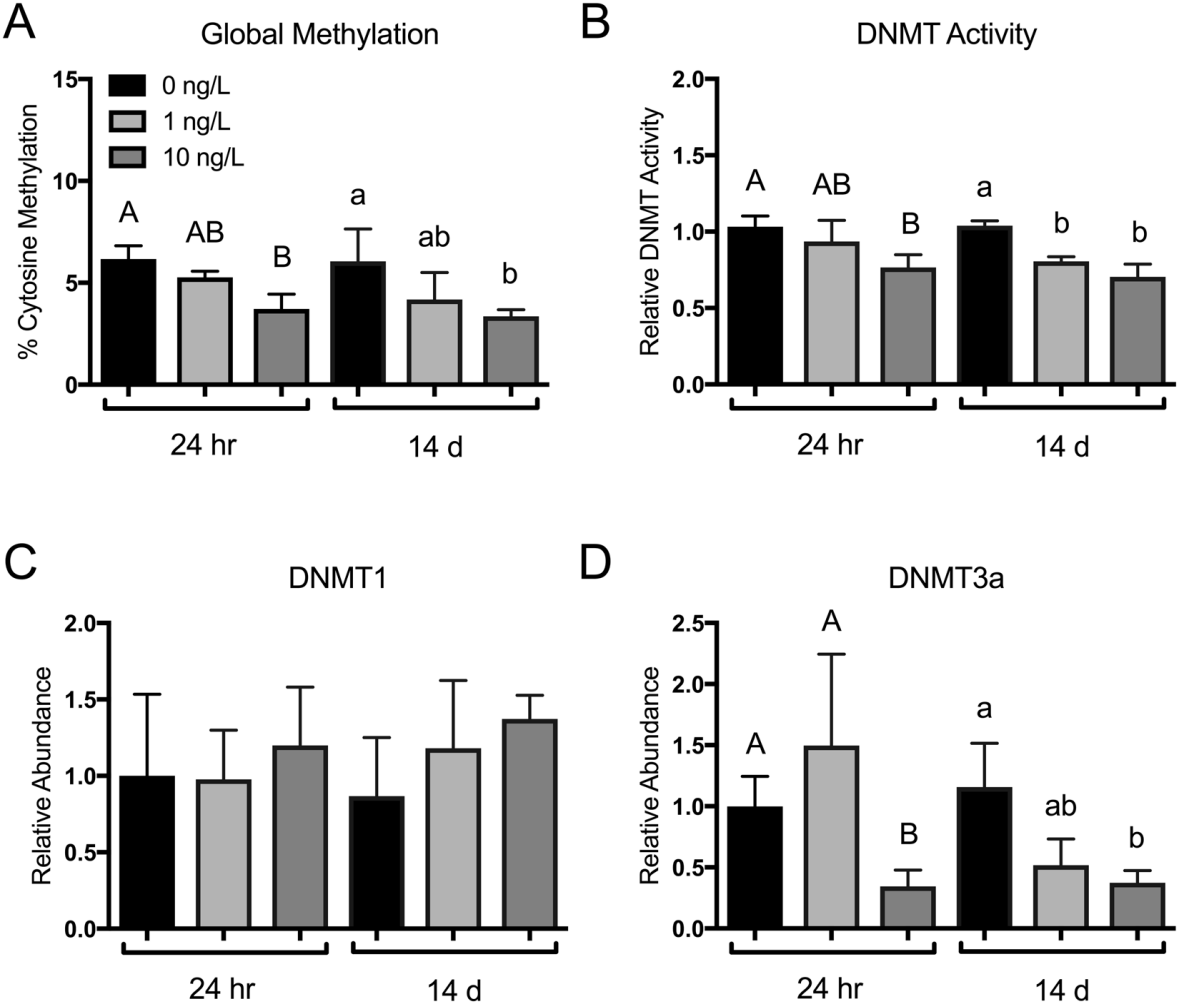
(**A**) Global liver methylation (% cytosine methylation), (**B**) DNMT enzymatic activity, and (**C**) DNMT1 and (**D**) DNMT3a relative transcript abundance in rainbow trout exposed to 0, 1, and 10 ng/L waterborne B[a]P after 24 h and 14 d. Bars that do not share a common letter are significantly different from the respective control during a given exposure time as determined by a 1-way ANOVA and Tukey’s post-hoc test (*p* < *0.05; n* = *9*).

Global DNA methylation was significantly down regulated in the liver after exposure to 10 ng/L B[a]P, independent of exposure time (Fig. 1A). This decrease in methylation was in part associated with a decrease in the activity of DNMTs (Fig. 1B). Although it should be highlighted that while there was a significant decrease in DNMT activity after a 14 d exposure of 1 ng/L, this only contributed to a non-significant decrease in % methylation at 14 d. Therefore dose is likely a significant driver of significant hypomethylation following B[a]P exposure. The nature of the commercially available kit quantified the activity of all DNMTs following nuclear extraction, as there is no kit available for specific activity of DNMT1 or DNMT3a in rainbow trout. However, following relative transcript abundance analysis of DNMT1 and DNMT3a, we effectively demonstrate a significant, near 75% reduction in liver DNMT3a transcript abundance under 10 ng/L B[a]P exposure, regardless of exposure time (Fig. 1D). There were no significant changes associated with DNMT1 transcript abundance (Fig. 1C). Therefore, it is likely that *DNMT3a* was a primary driver of the decrease in methylation activity and patterning in the liver of rainbow trout following B[a]P exposure.

Liver somatic index (1.32 ± 0.04) and condition factor (1.04 ± 0.01) calculated on samples fish fell within the expected range of standard animal health, and there were no detectable differences across tanks or treatments. Further, measurements of pH (8.22 ± 0.03), NH_4_ (1.17 ± 0.06 mg/L), NH_3_^+^ (0.04 ± 0.01 mg/L), and dissolved oxygen (10.34 ± 0.1 mg /L) were consistent with healthy housing conditions, and there were no detectable differences across tank replicates or treatments. Therefore, we conclude that the observed differences in global methylation, DNMT activity and transcript abundance were associated with the B[a]P treatment alone.

### Increased levels of a DNMT3A-targetting miRNA following B[a]P exposure

**Figure 2:**
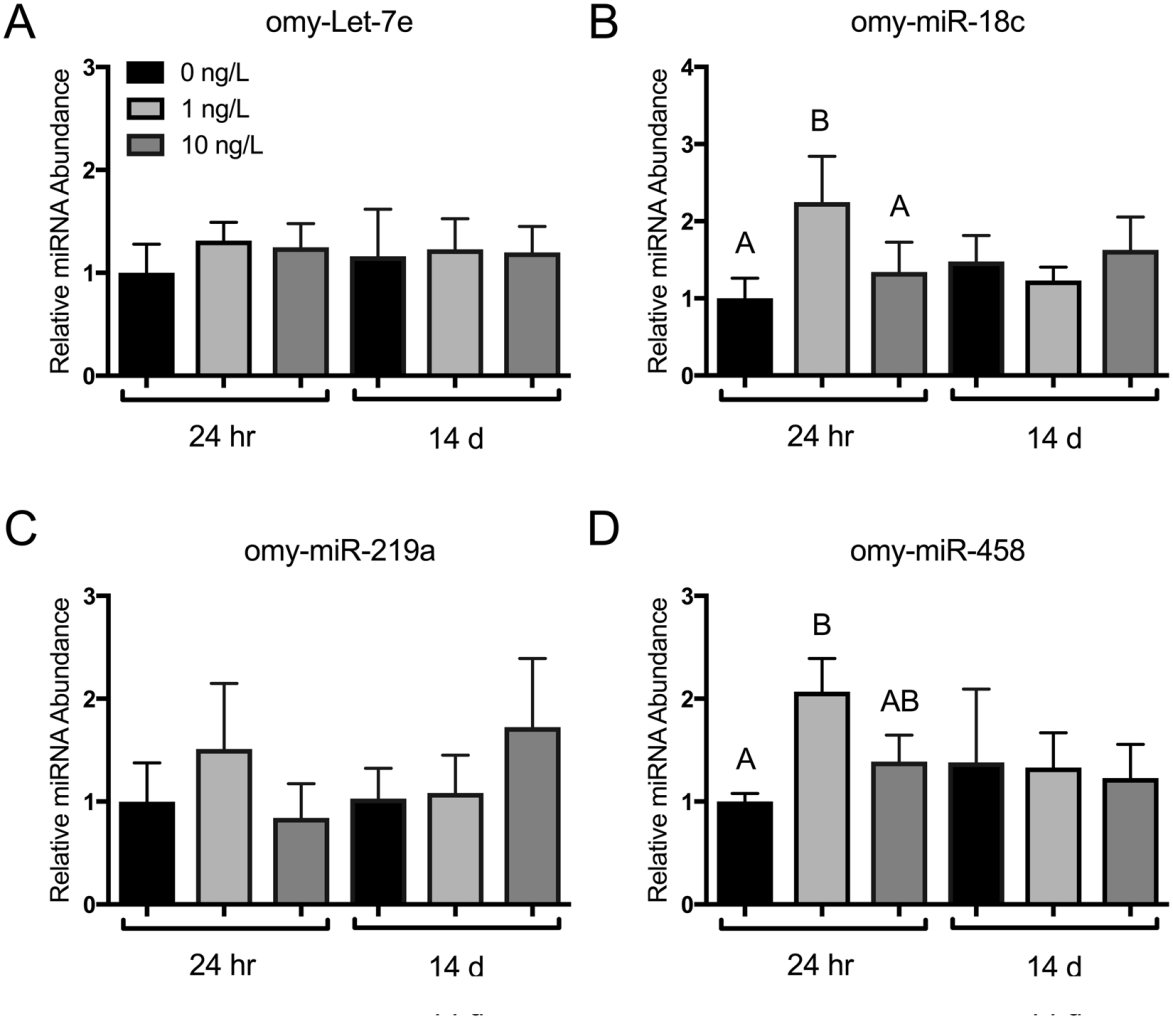
Relative abundance of liver microRNA predicted to bind to DNMT1 in rainbow trout exposed to 0, 1, and 10 ng/L waterborne B[a]P after 24 h and 14 d. (**A**) omy-let-7a, (**B**) omy-miR-18c, (**C**) omy-miR-219a, and (**D**) omy-miR-458 had a maximum likelihood *in silco* of targeting DNMT1. Bars that do not share a common letter are significantly different from the respective control during a given exposure time as determined by a 1-way ANOVA and Tukey’s post-hoc test (*p* < *0.05; n* = *9*). There was no correlation detected between significantly up-regulated miRNA at 24 h and DNMT1 relative transcript abundance.

**Figure 3:**
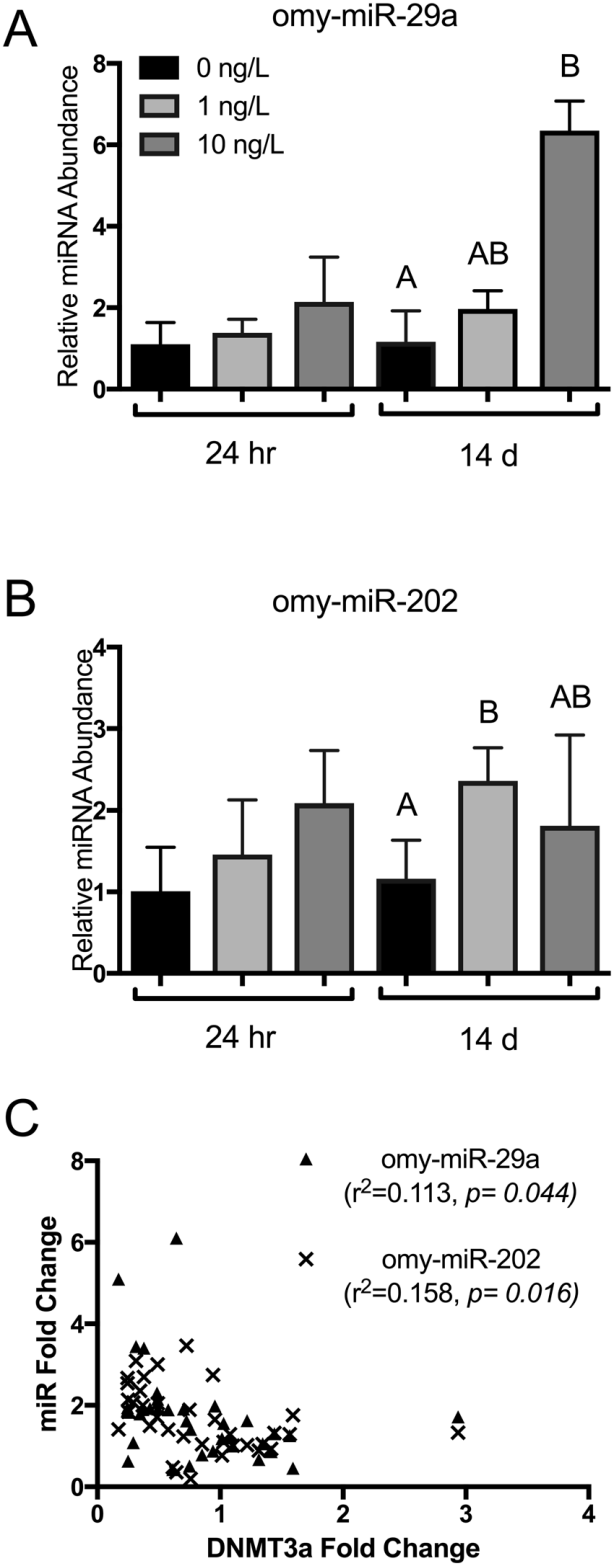
Relative abundance of liver microRNA predicted to bind to DNMT3a in rainbow trout exposed to 0, 1, and 10 ng/L waterborne B[a]P after 24 h and 14 d. (**A**) omy-miR-29a and (**B**) omy-miR-202 had predicted maximum likelihood *in silic*o of binding to DNMT1. Bars that do not share a common letter are significantly different from the respective control during a given exposure time as determined by a 1-way ANOVA and Tukey’s post-hoc test (p < 0.05; n = 9). There is a significant, inverse correlation (**C**) between relative miRNA abundance of both miRNA and their predicted DNMT3a target, wherein increased expression of these miRNA resulted in decreased relative abundance of DNMT3a. (Pearson’s correlation analysis; *p* < *0.05; n* = *9*).

Given that miR-29 has been widely implicated in the regulation of human DNMT3A^35–38^, we hypothesized that miR-29 may also be regulating *O. mykiss* DNMT3A in response to B[a]P treatment. Supporting this, we identified several miR-29 binding sites in the 3′UTR of *DNMT3A* (described in following section, and see Methods). To examine differential abundance of these miRNAs following B[a]P exposure, we performed quantitative PCR on the relative abundance of miR-29 and also several miRNAs predicted to target the 3′UTR of *DNMT1*, including omy-Let-7e, omy-miR-18c, omy-miR-219a, and omy-miR-458. MiRNA predicted to target *DNMT1* demonstrated no significant patterning associated with B[a]P exposure, although there was some fluctuations in omy-miR-18c and omy-miR-458 during the initial 24 h exposure to 1 ng/L B[a]P (Fig. 2B,D). Conversely, miRNA that were predicted to target *DNMT3a* (miR-29), demonstrated a significant increase in relative abundance that strongly correlated with the decrease in target abundance (Fig. 3). This was particularly true of omy-miR-29a, which displayed a 6-fold increase in relative abundance within the liver of trout following 10 ng/L B[a]P exposure (Fig. 3A). Pearson’s correlation analysis indicated a significance, inverse relationship between both omy-miR-29a and-202 and DNMT3a, which is expected if these miRNA target and repress relative abundance of DNMT3a (Fig. 3C). This relationship took all data points into account, covering both exposure and time.

### mir-29a binding sites are abundant in the DNMT3A 3′UTR and conserved across 500 million years of evolution

In order to investigate the molecular basis for miR-29 mediated regulation of DNMT3A following B[a]P exposure, we examined the 3′UTR of *DNMT3a* for putative miRNA binding sites. Based on available genomic contigs from the Genoscope Resource, a 1,143 bp 3′UTR of Dnmt3a was identified and annotated over the region 353436–354578 on *O. mykiss* genomic scaffold 64 in the NCBI (http://www.ncbi.nlm.nih.gov/nuccore/620599732). Further guided by comparison with the rainbow trout cDNA from the NCBI EST database (e.g., accession # BX868588), we identified a longer putative 3′UTR extending to 3,385 in length. The 3′UTR was followed by a poly-A tail as expected and is more consistent with the 3′UTR length (3,903 nt) of the reference zebrafish Dnmt3ab gene from the Ensembl database (accession # ENSDARG00000015566.1).

**Figure 4:**
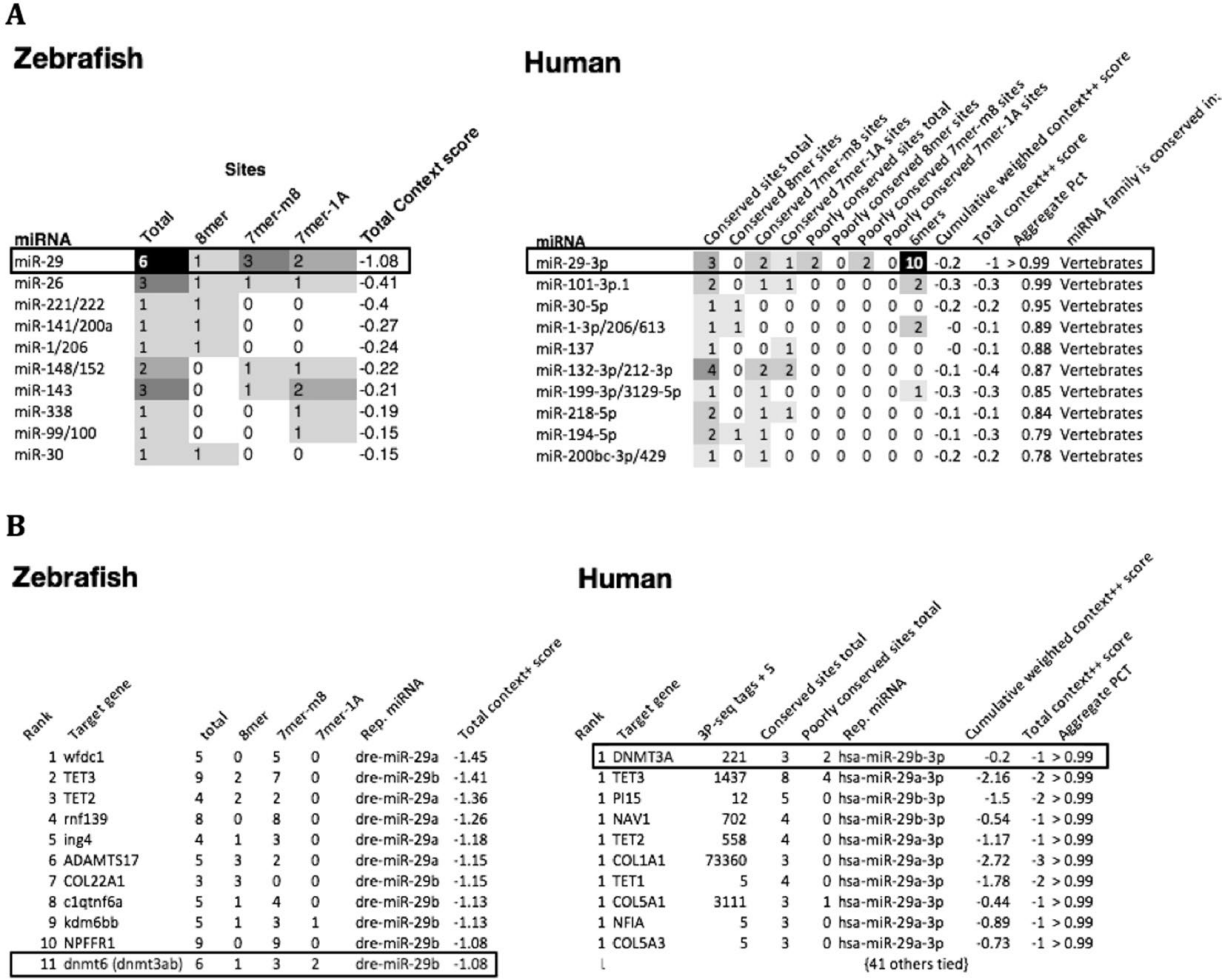
(**A**) miR-29 is the most abundant predicted microRNA binding site in Dnmt3a 3′UTRs in human and zebrafish. The predictions were obtained from TargetScanFish v. 6.2 and TargetScanHuman v. 7.1. Binding site predictions in Dnmt3a were ranked by Aggregate Pct (Friedman *et al*., 2009) score for human and Total Context score for zebrafish as PCT data were not available. (**B**) Dnmt3a is a top predicted genome-wide target of mir-29 in human and zebrafish. The predictions above were obtained from TargetScanFish v. 6.2 and TargetScanHuman v. 7.1. MiR-29 target predictions in were ranked by Aggregate PCT (Friedman *et al*.^21^) score for human and Total Context score for zebrafish as PCT data were not available.

**Figure 5:**
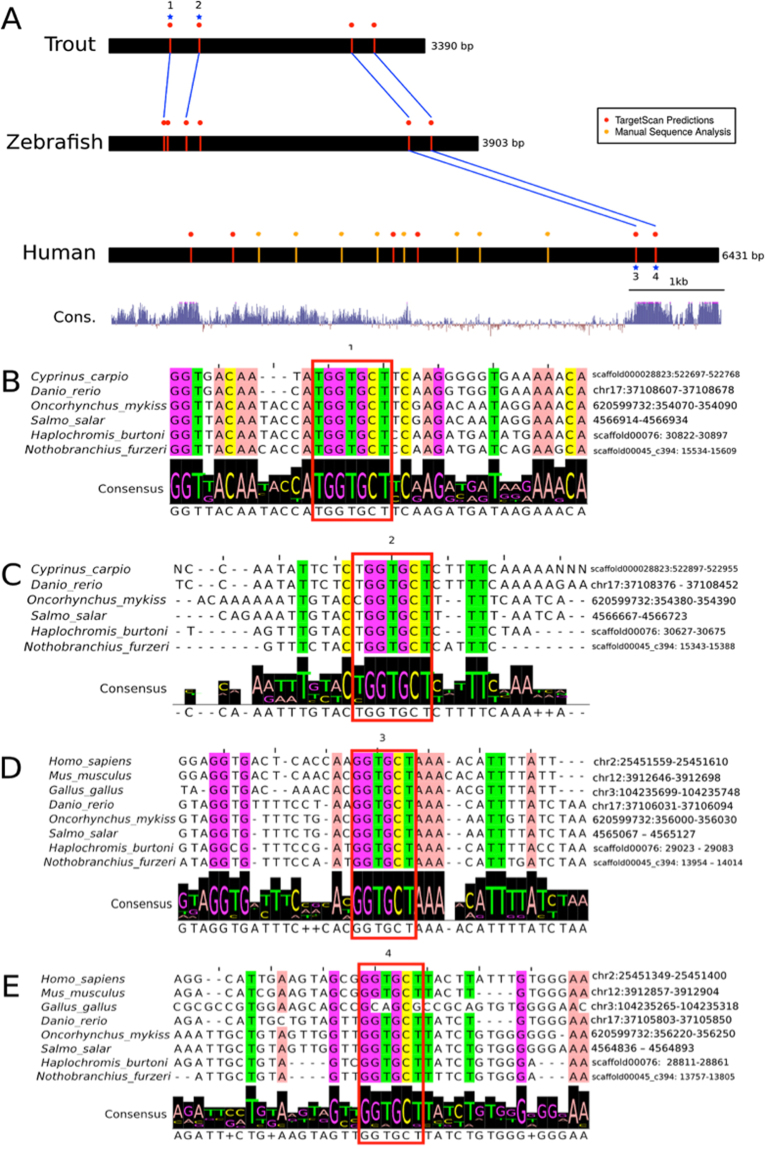
Identification of conserved mir-29 binding sites in 3′UTRs of Dnmt3a spanning mammals to fish. (**A**) A schematic of Dnmt3a 3′UTRs from trout, zebrafish, and human, with candidate matches to mir-29 binding sites shown by vertical lines. Binding sites were predicted using TargetScan as well as through additional manual k-mer searching of sequences. Starred sites are represented in (**B**–**E**). PhyloP scores from the UCSC multiz 100-way alignment are plotted below the human sequence indicating per-base evolutionary conservation. Two conserved mir-29 binding sites are located within a highly conserved block at the 3′ end of the 3′UTR. (**B**–**E**) Multiple sequence alignments of binding sites 1–4 labeled above. Binding sites 1 and 2 are conserved in fish while 3 and 4 are conserved in fish and mammals according to the Multiz 100-way vertebrate alignment from the UCSC genome browser.

Remarkably, the microRNA miR-29a was predicted as the most abundant miRNA targeting the *DNMT3a* 3′UTR in both zebrafish and humans (Fig. 4), and in addition we identified 4 putative miR-29a binding sites in the *O. mykiss* 3′UTR using TargetScan as well as through additional manual k-mer searching of sequences. Together, these predictions suggest conserved regulatory mechanism across vertebrates. To examine this further we examined the per-base evolutionary conservation of the *DNMT3A* region across vertebrates using the multiz 100-way alignment in the UCSC Genome Browser. PhyloP scores measuring the degree of evolutionary conservation are plotted below the human sequence (Fig. 5). Two conserved mir-29 binding sites are located within a highly conserved block at the 3′ end of the 3′UTR. Several binding sites (172; Fig. 5) were consistently conserved in fish species, while binding sites 3 & 4 were conserved across fish to humans. The conserved miR-29 binding sites and surrounding sequence in the 3′UTR of *DNMT3A* across vertebrates is striking as it suggests an ancient mechanism of miR-29 mediated DNMT3A regulation that originated within the common ancestral tetrapod approximately 500 millions years ago.

## Discussion

Our research strongly substantiates the importance of miRNA regulation, especially miR29a, on the epigenome. MiRNAs act as the interface between environment and the expression of genes, regulating changes in order to combat exposures to environmental stressors. Numerous environmental studies examining changes on the miRNA transcriptome have demonstrated a clear association between environmental factors, such as diet, drugs, and pollutants, with changes in miRNA abundance^39^. Moreover, this research exemplifies the importance of strict miRNA regulation on the genome by demonstrating miRNA dysregulation in response to environmental pollutants and its global effects on the genome. As this research demonstrates, miR29a acts as a mediator between the environment and the genome by regulating a key methylation enzyme, DNMT3a. As a result, miR29a must also undergo strict regulation to prevent aberrant DNMT3a expression, global DNA hypomethylation, and ultimately genomic instability. The importance of miR29a regulation is further exemplified in its evolutionary conservation. Our research shows that there are 4 miR29a binding motifs in the 3′UTR of the DNMT3a transcript that are highly conserved across fish species. Furthermore, binding motifs 3 & 4 are conserved throughout vertebrate evolution, spanning fish to humans. It is also important to note that while the human DNMT3a 3′UTR has diverged to lose the highly conserved binding motifs 1 & 2, there is an overall increase in predicted miR29a binding sites in humans compared to fish (Fig. 5). Previous work has shown that a very low proportion (<10%) of miRNA target sites are conserved from mammals to fish^23^. The deeply conserved miR29a binding sites in DNMT3a 3′UTRs identified in this study fall into this category, and suggest that miR29a is one of the key miRNA regulators of epigenetic change by acting upon DNMT3a. Conversely, other non-essential miRNA-binding motifs of DNMT3a may be lost throughout evolution, or may have diverged in parallel with the sequences of their miRNAs. Taken together, our data show that carcinogenic B(a)P plays a significant role in altering the epigenome of rainbow trout. Acute and chronic B(a)P exposures resulted in the dysregulation of miR29a. Ultimately, these exposures led to potentially detrimental hypomethylation of genomic DNA, a hallmark of genomic instability and cancer, through a miR29a-DNMT3a regulatory pathway that is conserved across 500 million years of evolution. Moreover, bioinformatics analysis provides further evidence that miR29a is a major regulator of epigenetic methylation enzymes and its regulation is an interface between the environment and the genome.

DNA methyltransferases are of utmost importance in maintaining and establishing methylation patterns in most organisms^40^. During development, genomic methylation reprogramming occurs, causing the loss of certain parental methylation patterns. After various developmental time points, specific methylation patterns are restored, which has broad developmental potential during differentiation^41^. It has been effectively demonstrated in several fish species numerous environmental and developmental factors affect methylation patterning, such sex reversal in fish^42^, metamorphosis in lamprey^43^, adaptive responses to salinity in brown trout^44^, and contaminant exposure in eels^45^. Common amongst these studies is the disruption of the methylation patterning driven by changes DNMT activity. While DNMT function is critical in early development, its dysregulation has been studied in relation to numerous pathologies, including a variety of cancers^46^. In cancer, global DNA hypomethylation, a hallmark of genomic instability, is the most common epigenetic phenotype that is observed^47^. As a result, it is not surprising that inactivation or loss of DNMT protein expression plays a causal role in promoting global DNA hypomethylation and subsequent tumorigenesis^47^, evidence that supports the hypomethylation pattern we have demonstrated in trout exposed to B[a]P (Fig. 1A). With respect to DNMT3a, somatic mutations have been identified as causal factors in myeloid leukemia^48,49^ and hematological malignancies^50^; however, discrepancies in all DNMT isoforms are associated with their own class of cancers. Cumulatively, the study of the strict regulation of DNMTs in genomic stability, development and physiology cannot be understated.

Our analysis of DNMTs in this teleost model provided a significant contribution to our knowledge of epigenetic regulation evolutionarily. Firstly, we were able to show a direct relationship between an environmental carcinogen and dysregulation of a major *DNMT* miRNA regulator. Secondly, through analyzing the 3′UTR across species, there was significant miR29a target site conservation, outlining the importance of this regulatory pathway. These results are further corroborated in acute myeloid leukemia studies, where induction of miR-29b resulted in a marked reduction of *DNMT3a/b* mRNA and global DNA hypomethylation^51^. In conjunction, our evolutionary analysis of this regulatory pathway is substantiated in mammalian models demonstrating the translatability of this regulatory pathway across species. This research further outlines the importance of understanding DNMT regulation upon environmental factors with respect to disease etiology.

We have shown a significant evolutionary conserved pathway, in which miR-29a regulates *DNMT3a* expression. Furthermore, similar results were previously elucidated in acute myeloid leukemia studies demonstrating that exogenous miR-29b expression downregulated *DNMT3a/b* mRNA in human cancer cell lines^51^. Cumulatively, these studies have clinical significance to assess environmental exposures and provide a physiological marker for epigenetic dysregulation. For instance, to examine the effects of other environmental pollutants on the epigenome, one could examine miR-29a levels and subsequent DNA methylation in exposed organisms. Furthermore, since miR-29a dysregulation and DNMT deficits are associated with many cancers, miR-29a could be measured as an early detection tool for genomic instability resulting from DNA hypomethylation.

Additionally, the exposure to B(a)P caused an increase in miR-29a and miR-202, which is mirrored in a variety of cancers^51,52^. In miR-29 transfection studies, global DNA methylation, *DNMT1, -3a* transcript, and protein levels showed statistically significant decreases^53^. Moreover, the same results were observed in this study when miR-29a abundance increased in response to B(a)P exposure. One exception between results herein is that *DNMT1* levels did not show a decrease in response to change in miR-29a, however protein quantification would be required to confirm this discrepancy. The research by Fabbri *et al*.^53^ also stresses the importance of protein quantification of DNMT1 and DNMT3 by further confirming relationships between candidate miRNA expression changes and their downstream DNMT targets. Furthermore, this research compliments recent literature by demonstrating that other miRNA exhibit effects on the genome and that environmental stressors trigger expression changes in these miRNA. In toxicological studies in trout embryos exposed to B(a)P, morphology and developmental defects were observed^54^. Depletion of yolk sac, kyphosis, lack of cellular pigmentation and defects or lack of eyes were observed in trout exposed to B(a)P^54^. From our results, some of these observed abnormalities could be explained by epigenetic changes through DNMTs, however further research would be required. This research establishes that miRNA act as a mediator between the environment and epigenetics through DNMTs, which is further observed by an evolutionarily conserved regulatory relationship between miR-29a and DNMTs in mammals and teleosts.

## Methods and Materials

### Animals and exposure

Rainbow trout (*O. mykiss; immature, mixed sex, 25.2* ± *0.4* *g*) were obtained from Silver Creek Aquaculture (Erin, ON) and acclimated for at least 2 weeks in 200 L aerated tanks with constant water flow at 13 °C and a 12 h light: 12 h dark photoperiod prior to experimentation. Although there were mortalities that occurred during the course of the experiment, these were likely related to the general condition of the animal upon arrival, as mortalities were noted in all tanks (1–2 per treatment; data not shown). Fish were then moved into separated aquariums and allowed to acclimate for 1 week prior to the start of the exposure. For each of the exposures, there were 9 fish placed in individual tanks, and tanks were replicated 3 times for each exposure regime (0 ng/L; 1 ng/L; 10 ng/L B[a]P). All tanks were dosed continuously in a flow through manner for the duration of the experiment. Animals were monitored throughout the duration of the treatment. After 24 h and 14 d, fish were terminally anesthetized with an overdose of buffered tricaine methanesulfonate (0.5 g/L MS-222; 1 g/L HCO_3_, Sigma), weighed, whole livers were removed which were immediately freeze-clamped in liquid nitrogen, and stored at −80 °C. All liver tissue was first ground to a fine powder under liquid nitrogen, weighed into aliquots, and stored at −80 °C until processed to reduce potential issues with thawing. For this experiment, only 3 fish from each tank replicate were used, with the remaining fish repurposed for a separate experiment. Therefore, the total number of fish sampled per treatment was 9. All experimental procedures followed strict ethical guidelines and were approved by the University of Waterloo animal care committee (AUPP #14–15).

### Global methylation and DNMT activity

For quantification of global methylation, genomic DNA was first extracted from 25 mg of frozen, powdered liver tissues using the DNeasy Blood & Tissue Kit (Qiagen) following the manufacturers instructions. DNA was quantified on a SpectraMax 190 spectrophotometer (Molecular Devices) using a SpectraDrop microvolume microplate (Molecular Devices). Once extracted, DNA was stored at −20 °C until required. 100 ng of DNA was used to assess the global methylation in the liver of rainbow trout. Global methylation was determined via MethylFlash™ methylated DNA colorimetric quantification kit (Epigentek) following the manufacturers explicit instructions. Global methylation is expressed as a percentage of cytosine methylation as determined by the provided standard curve. Total DNMT activity was determined using the EpiQuik™ DNMT activity/inhibition assay ultra kit (Epigentek) following the manufacturer’s protocol. Briefly, nuclear proteins, including DNMTs, were extracted from 20 mg of frozen liver tissue using the EpiQuik™ nuclear extraction kit (Epigentek). 10 μg of nuclear extract was used for each liver sample and assessed using the DNMT activity kit. Total DNMT activity is presented as relative activity compared to the control.

### Bioinformatic identification of Dnmt3ab and DNMT1 3′UTRs in the rainbow trout genome

To identify the Dnmt3a gene and associated 3′UTR within the recently published rainbow trout genome^31^, we retrieved and analyzed raw genomic contigs from the Genoscope Resource (http://www.genoscope.cns.fr/trout/). TBLASTN searches using zebrafish Dnmt6 (uniprot identifier B3DGZ8) yielded the gene identifier GSONMT00080191001 as the top-scoring hit (*E*-value = 0). BLAST searches against the NCBI nr database with GSONMT00080191001 as the query yielded Salmon Dnmt3a with 99% identity (*E*-value = 0), confirming GSONMT00080191001 as *Dnmt3a* from rainbow trout. TBLASTN searches using zebrafish (Dnmt1) (uniprot identifiers B3DKL6 and B3DKL7) yielded the gene identifier GSONMT00021648001 as the top-scoring hit (*E*-value = 0). BLAST searches against the NCBI nr database with GSONMT00021648001 as the query yielded Salmon Dnmt1 isoforms 1–4 as the top queries with 98% identity (*E*-values = 0).

### DNMTs and microRNA quantification

3′UTRs of DNMT1 and DNMT3a were then run through mirbase.org. Candidates were chosen and scored based on greatest base pair matching and the greatest number of binding sites. Candidates were also chosen using targetscanfish.com, which analyzes the DNMT1 and DNMT3a 3′UTR in related zebrafish transcripts to determine miRNA regulatory candidates. DNMT1 candidates were omy-let-7e, omy-miR-18C, omy-miR-219a, and omy-miR-458, while the ideal candidates for DNMT3a were omy-miR-29a and omy-miR-202. Additionally, small nucleolar RNA U6 (snoU6) sequence was extracted from the trout genome database to serve as an endogenous control gene, to normalize miRNA quantification data, as this has been identified as a suitable reference RNA target for normalization of miRNA using quantitative RT-PCR^55^. Primer information is listed in Table 1, and sequences validated through the recently published rainbow trout miRNA transcriptome^32^. Total RNA, including microRNA, was extracted from 25 mg frozen liver tissue (n = 9) using the Qiagen miRNeasy kit (Qiagen). Total RNA and microRNA concentrations and purity were validated spectrophotmetrically using the SpectraDrop microvolume microplate on a Spectramax 190 (Molecular Devices). Additionally, a small aliquot of each sample was run electrophoresed on an agrose gel to validate RNA integrity. First-strand cDNA for quantification of DNMT1 and DNMT3a transcripts was synthesized using the QuantiTect Reverse Transcription kit (Qiagen) starting with 1 µg of RNA. Relative quantification of DNMT1 and DNMT3ab, using the QuantiTect SYBR green kit (BioRad), was assayed on the CFX96 real-time system (BioRad). Each reaction contained 5 uL SYBR mix, 1 µL of each forward and reverse primer (0.3 μM), 1 µL of 5x diluted cDNA template, and 2 µL RNase/DNase H_2_O. Cycling conditions were: 15 min initial activation at 95 °C followed by 40 cycles of 95 °C for 15 s, 60 °C for 30 s, and 72 °C for 30 s. To account for differences in amplification efficiencies between different cDNA, standard curves were constructed for each target transcript using serial dilutions of pooled cDNA. To account for differences in cDNA production and loading, all samples were normalized to the abundance of the house-keeping gene elongation factor-1a, which did not change over the experimental treatments and is a common housekeeping normalization transcript for mRNA. Relative abundance data was calculated using the 2^ΔΔ−CT^ method^56^, as all standard curves had an efficiency of 100%. Both RNase/DNase-free H_2_O and non-reverse transcribed RNA were assayed on each plate to ensure that no contaminating DNA was present in the reagents used. Further, melt-curve analysis was performed on all samples to ensure only one product was formed.

**Table 1:** Primer sequences used for qPCR quantification of the relative abundance of miRNA and DNMT1 and 3a transcripts from the liver of rainbow trout exposed to B[a]P.

miRNA/transcript	Fwd	Rev
omy-Let7e-3p	CTATACAATCTACTGTCTTTCC	Universal Primer (Qiagen)
omy-miR-18c	TAAGGTGCATCTTGTGTAGTTA	Universal Primer (Qiagen)
omy-miR-219a	TGATTGTCCAAACGCAATTCTT	Universal Primer (Qiagen)
omy-miR-458	GCAGTACCATTCAAAGAGCTAT	Universal Primer (Qiagen)
omy-miR-29a	TAGCACCATTTGAAATCGGTTA	Universal Primer (Qiagen)
omy-miR-202	TTCCTATGCATATACCGCTTT	Universal Primer (Qiagen)
Sno-U6	GGCTTCGGCAGCACATATAC	Universal Primer (Qiagen)
DNMT1	CCTGACCGATTCTACTTCCTTG	CTTCCCTTTGCCTTTACCTTTG
DNMT3a	CAGCGACAAGAGGGACATC	AGTCAAGGGTCTGTTCATGC
EF1a	TCCTCTTGGTCGTTTCGCTG	ACCCGAGGGACATCCTGTG

For first-strand synthesis of miRNA, the miScript II RT kit (Qiagen) was used, with a starting amount of 250 ng RNA for each sample. Relative quantification of all *in silico* predicted miRNA was performed using the Qiagen miScript SYBR green kit following the manufactures instructions to a final volume of 25 µl per reaction. The reaction conditions were as follows: 15 min initial incubation at 95 °C followed by 40 cycles of 95 °C for 15 s, 55 °C for 30 s, and 72 °C for 30 s. This was followed by a melt-curve analysis to ensure only one product was formed. Standard curves were formed for all targeted miRNA, and all validation steps were carried out as above to ensure proper efficiency and no contamination was detected in each sample. Relative abundance was calculated using the 2^ΔΔ−CT^ method^56^. For relative abundance calculations, the house-keeping small RNA U6 was used as this is common for normalization of miRNA in quantitative RT-PCR, which did not change over the experimental treatments.

### Dnmt3ab 3′UTR alignments and microRNA binding site predictions

To construct an alignment of *Dnmt3a* 3′UTRs from fish, BLASTN searches were performed against the Euteleostomi subset of the NCBI database with zebrafish *Dnmt6* 3′UTR as the query. Homologous sequences from fish were selected from the BLAST hits, all with *E*-value < 0.01, and verified as 3′UTRs from *Dnmt3a* orthologs. Sequences included *Dnmt3a* 3′UTRs from the following species and genome identifiers: Common carp genome (GCA_000951615.1); *Danio rerio*: danRer7; Salmon genome: ICSASG_v2v (GCF_000233375.1); *Haplochromis burtoni*: AstBur1.0 (GCF_000239415.1); *Nothobranchius furzeri*: GRZ genome assembly (GCF_001465895.1); *O. mykiss* genomic scaffold, scaffold_64; *Homo sapiens*: Hg19; *Mus musculus*: mm10; *Gallus gallus*: galGal4; *Danio rerio*: danRer7 (for UCSC MAF). These sequences were subsequently aligned using MUSCLE version 3.5^57^ with default parameters.

Using the Multiz-100 way alignment from the UCSC browser, the fish *Dnmt3a* 3′UTR alignment was then merged with orthologous sequences from additional non-fish vertebrate species including mammals. Sections of the Multiz-100way alignment were taken from UCSC table browser (hg19) and converted using ‘MAF to FASTA’ from Galaxy^58^; human, mouse, chicken and zebrafish sequences were extracted for alignment. The zebrafish sequence was used to align the fish 3′UTR MSA to the subset Multiz alignment via Seaview, with sequence conservation and logos generated using Jalview (coloured with the ‘Zappo’ colouring scheme at an 85% identity threshold). PhyloP scores from the UCSC vertebrate Multiz 100-way alignment were also retrieved and mapped onto the alignment. TargetScanFish v. 6.2 and TargetScanHuman v. 7.1 were then used to predict microRNA binding sites in zebrafish *Dnmt6* (*Dnmt3ab*) and human *Dnmt3a*, respectively. Additional exact matches to mir29 6-mers (GGTGCT) were also identified in the human 3′UTR sequence regardless of their position (no additional k-mer matches were detected in fish 3′UTRs), and evolutionarily conserved binding sites identified by visual inspection. Sequence logos were generated with Jalview version 2 (http://www.jalview.org/).

### Statistical analysis

All data are presented as a mean ± SEM. Graphical and statistical analysis was carried out using Prism 6 (GraphPad Software). Statistical differences were determined between all treatments within a given time period using a one-way ANOVA followed by a Tukey’s multiple comparison test, as the goal was to examine the acute dose response and chronic dose response, but not the difference between time. Hence, we did not use 2-way ANOVA. However for simplicity, we combined the 24 h and 14 d data on the same graphs. Correlation analysis for the inverse relationship between relative abundance of miRNA and DNMTs was performed using a Pearson’s correlation. The level of significance for all tests was set a *p* < *0.05*. All data passed test of variance and normality, and the analysis was performed on the raw data.

### Data Availability

The datasets generated during and/or analyzed during the current study are available from the corresponding author on request.
